# Neuronal signalling of zinc: from detection and modulation to function

**DOI:** 10.1098/rsob.220188

**Published:** 2022-09-07

**Authors:** Chen Zhang, Anna Dischler, Kaitlyn Glover, Yan Qin

**Affiliations:** Department of Biological Sciences, University of Denver, Denver, CO 80210, USA

**Keywords:** neuronal, signalling, zinc, sensor

## Abstract

Zinc is an essential trace element that stabilizes protein structures and allosterically modulates a plethora of enzymes, ion channels and neurotransmitter receptors. Labile zinc (Zn^2+^) acts as an intracellular and intercellular signalling molecule in response to various stimuli, which is especially important in the central nervous system. Zincergic neurons, characterized by Zn^2+^ deposits in synaptic vesicles and presynaptic Zn^2+^ release, are found in the cortex, hippocampus, amygdala, olfactory bulb and spinal cord. To provide an overview of synaptic Zn^2+^ and intracellular Zn^2+^ signalling in neurons, the present paper summarizes the fluorescent sensors used to detect Zn^2+^ signals, the cellular mechanisms regulating the generation and buffering of Zn^2+^ signals, as well as the current perspectives on their pleiotropic effects on phosphorylation signalling, synapse formation, synaptic plasticity, as well as sensory and cognitive function.

## Graphical abstract

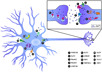

## 1. Introduction

As the second most abundant trace element after iron, zinc has the highest concentration in the brain (6–95 µg g^−1^) [1–3], and is essential for brain function. Prenatal zinc deficiency during the critical period of rapid brain growth in fetuses causes irreversible damage to neural development [4–10], impairs learning and memory [11], and leads to development of autism-like behaviour [12]. Such nutritional zinc deficiency impedes neurogenesis, neuronal migration and synaptogenesis during brain development [13]. Zinc deficiency in adults has been clinically linked to psychological disorders such as depression [14,15], attention-deficit/hyperactivity disorder (ADHD) [16,17], and autism [18], as well as neurodegenerative diseases such as Parkinson's disease [19–21].

Zinc's importance in the brain is due to its high protein binding capacity [22], which serves critical roles in the structural stability, catalytic activity and regulatory function of thousands of proteins. In the past few decades, the focus of biological study on zinc has expanded from its structural and catalytical roles to regulatory function and its signalling roles have been extensively investigated, especially in the immune system and central nervous system [23–25]. This review focuses particularly on neurons, the fundamental units that generate electrical and chemical signals in the brain.

Neuronal signalling is partially mediated by free or labile zinc (referred to as Zn^2+^ ) via intracellular and intercellular signalling pathways. Inside neurons, the majority of zinc is present in a protein-bound state, leaving only a subnanomolar concentration of labile Zn^2+^ in the cytosol [26–29]. Intracellular Zn^2+^ homeostasis is efficiently maintained at stable levels with buffering and muffling mechanisms involving Zn^2+^ transporters, metallothioneins and metal-responsive transcription factor-1 [26,27,30,31]. A high concentration of Zn^2+^ is stored in the synaptic vesicles of certain neurons [28,29], which can be released into the synaptic cleft and act as intercellular signalling molecules when neurons are activated by physiological stimuli [31,32]. In addition, cytosolic Zn^2+^, via liberation from intracellular stores, can mediate a series of intracellular signalling events. In this review, we will provide an overview of neuronal Zn^2+^ signalling, both intracellularly and intercellularly. We will first summarize the fluorescent sensors that have been used to illuminate Zn^2+^ signals within subcellular compartments of neurons and synapses. Next, we will discuss the current understanding about synaptic Zn^2+^ signalling in the brain. We will then move onto discussing different players that modulate intracellular Zn^2+^ signals, followed by a discussion of the targets and function of Zn^2+^ in intracellular signalling pathways.

## 2. Detection of neuronal Zn^2+^ signals

The prerequisite of a signalling molecule is that its concentration can fluctuate alongside physiological events. Fluorescent sensors, along with time-lapse fluorescence microscopy, have been enormously instrumental in revealing spatio-temporal dynamic changes of Zn^2+^ signals in neurons and brain tissues. Fluorescent Zn^2+^ sensors involve a Zn^2+^-binding unit with one or two fluorescent components so that the sensors can display changes in spectral properties in response to the binding of Zn^2+^ ions. These tools include synthetic small molecule sensors that are constructed by organic chemistry and genetically encoded sensors that are constructed by molecular protein engineering. Cell impermeable small molecule sensors can be used to detect extracellular Zn^2+^ signals in brain slices, while detection of intracellular Zn^2+^ can be achieved by the addition of an ester moiety, which allows sensors to cross the plasma membrane into cells, followed by cleavage via intracellular esterases [33,34]. However, incomplete hydrolysis of these esters can cause nonspecific localization of small molecule sensors to subcellular compartments [35]. Small molecule sensors generally display a very large dynamic range and are easy to use in comparison to genetically encoded sensors. Genetically encoded sensors, on the other hand, are superior in spatio-temporal detection because they can be precisely targeted to specific cell types and subcellular compartments [36–38]. Genetically encoded sensors are ideal for long-term imaging due to their ability to be retained in cells for days to weeks [36,39]. Please see these review papers [38,40,41] for a comprehensive overview of different types of Zn^2+^ sensors. Here we will focus on the sensors that have been successfully used to detect dynamic changes in Zn^2+^ signals in neuron cultures and brain slices (table 1).

**Table 1.** List of some sensors used to measure and detect neuronal Zn^2+^ signals.

localization	name	dissociation constant (*K*_d_)	application	references
cytosol	ZP1	0.7 nM	hippocampal slices	[35,42–44]
			primary neurons	
	ZP3	0.7 nM	hippocampal neurons and slices	[30,45]
	ZnAF-2DA	2.7 nM	hippocampal slices	[46]
	FluoZin-3 AM	15 nM	primary neurons	[33,47–51]
			hippocampal slices	
	GZnP3	1.3 nM	primary neurons	[52]
mitochondria	RhodZin-3	65 nM	primary neurons	[39,53–57]
			PC12 cells	
	Mito-ZapCY1	1.6 pM	*C. elegans* PVD neurons	[58,59]
lumen of synaptic vesicles and acid compartments	TSQ	155 nM–48 µM	hippocampal slices	[35,60–66]
	Zinquin	620 nM	primary neurons	[60,64–67]
	SpiroZin-2	3.6 nM	hippocampal slices	[51,68]
extracellular regions (synaptically released Zn^2+^)	FluoZin-3	15 nM	hippocampal slices	[33,69,70]
	ZP4	0.65 nM	hippocampal slices	[71–73]
			primary neurons	
	ZnAF-2	2.7 nM	hippocampal slices	[34,42,46,74–77]
	NewPort Green DCF	1 μM	hippocampal slices	[78–81]
	LZ9	0.57 nM	coronal brain slices containing dorsal cochlear nucleus	[82–84]

Steady-state Zn^2+^ concentration in the cytoplasm of mammalian cells is maintained at a subnanomolar range (100 pM–1 nM) [33,85–87]. To detect cytosolic Zn^2+^ signals changing from baseline concentrations to high nanomolar concentrations, we need sensors with nanomolar sensitivity which is determined by both binding affinity and dynamic range. A number of cell permeable small molecule sensors with nanomolar affinity have been reported including ZnAF-2DA (*K*_d_: 2.7 nM) [88], ZP1 (Zinpyr-1, *K*_d_: 0.7 nM) [89], ZP3 (*K*_d_: 0.7 nM) [45], ZP4 (*K*_d_: 0.65 nM) [71] and FluoZin-3 acetoxymethyl (AM) ester (*K*_d_: 15 nM) [47]. Among these sensors, FluoZin-3 AM is one of the most popular sensors used to detect cytosolic Zn^2+^ signals because it displays large response to Zn^2+^ [47]. The fluorescence of FluoZin-3 is not affected by other cations such as Ca^2+^ and Mg^2+^, which allows simultaneous recording of Zn^2+^ and Ca^2+^ by using FluoZin-3 alongside the Ca^2+^ dye Fura Red [48], Fura-2FF [49,50] or Fura-6F [50] in primary cultured neurons and hippocampal slices. FluoZin-3 also has low sensitivity to changes in pH, with its signal remaining unchanged from pH 6 to pH 9 [48,90]. FluoZin-3 has successfully been used with pH sensor pHrodo Red to record dynamic changes in Zn^2+^ and pH simultaneously in cultured hippocampal neurons [48]. One limitation of using small molecule sensors to detect cytosolic Zn^2+^ in neurons is that their nonspecific subcellular localization generates variable fluorescence between the cytosol and bright punctate compartments, making it difficult to interpret the subcellular locations of Zn^2+^ signals. Genetically encoded sensors with more specific subcellular localization allow distinguishing Zn^2+^ signals coming from various subcellular compartments. For example, GZnP3 (*K*_d_: 1.3 nM) has been used to show that endolysosomal vesicles can release Zn^2+^ into the cytosol in hippocampal neurons [52].

Measurement of Zn^2+^ in the mitochondrial matrix by different sensors has determined that mitochondrial Zn^2+^ concentration is several orders of magnitude lower than the cytosol, approximately 0.2–300 pM [58,85,91–94]. RhodZin-3 (*K*_d_: 65 nM) is the first reported mitochondrial Zn^2+^ sensor, which has a 75-fold fluorescence change from quenched N,N,N′,N′-tetrakis(2-pyridinylmethyl)-1,2-ethanediamine (TPEN) to saturated Zn^2+^ levels *in vitro* [53,54]. The positive charges carried by RhodZin-3 allow it to follow the electrical gradient and accumulate in the mitochondria [54]. RhodZin-3 has been used to record increases in mitochondrial Zn^2+^ following ischemia [53], N-ethylmaleimide treatment [55] or A*β*42 incubation [39,56] in primary cortical and hippocampal neurons. Mitochondrial Zn^2+^ was also detected in the neuron-related PC12 cells using RhodZin-3 [57]. However, due to its positive charge, RhodZin-3 signals are reduced in mitochondria when the mitochondrial membrane is depolarized, which limits its proper localization [39,56]. Other small molecule Zn^2+^ sensors targeted to mitochondria include DA-ZP1-TPP [95] and ZP1BG which requires co-transfection with mitochondrial-targeted alkylguaninetransferase [96], but these probes have not been widely used in live cells. Three genetically encoded mitochondrial Zn^2+^ sensors based on fluorescence resonance energy transfer (FRET) have been created with various binding affinities: mito-ZapCY1 (*K*_d_: 1.6 pM, [58]), mito-eCALWY-4 (*K*_d_: 60 pM, [92]), and mito-eZinCh-2 (*K*_d_: 5–10 pM, [91]). These ratiometric probes are useful in quantification of mitochondrial Zn^2+^ concentrations. For example, mito-ZapCY1 has been used to measure mitochondrial Zn^2+^ in *C. elegans* posterior ventral dorsal (PVD) neurons [59]. Mitochondrial intermembrane space (IMS) is separated from mitochondrial matrix by the inner mitochondrial membrane, where oxidative phosphorylation occurs. A single fluorescent protein-based Zn^2+^ sensor, GZnP2, has been targeted to the mitochondrial matrix and the IMS [85], demonstrating that the concentration of labile Zn^2+^ in the IMS is 100 pM, revealing differences in Zn^2+^ concentration across inner mitochondrial membrane by three orders of magnitude [85].

Synaptic vesicles are unique secretory organelles found in neurons, which are categorized by the different neurotransmitters they contain. High concentrations of Zn^2+^ are concentrated in the synaptic vesicles of certain glutamatergic neurons [29,31,97–100], but also in some glycinergic and GABAergic neurons in neocortex [101,102], hippocampus [100,103–111], amygdala [31,107], auditory brainstem [112,113] and spinal cord [114,115]. The vesicular Zn^2+^ concentrations are estimated to be in the high nanomolar to low millimolar range [11,29,98,109,116–121]. This pool of Zn^2+^ inside the synaptic vesicles was visualized by small molecule sensors such as TSQ (*K*_d_: 155 nM to 48 µM), which is a quinoline-based membrane-permeable sensor [60,61]. TSQ is lipophilic making it ideal for measuring vesicular Zn^2+^ in brain tissue [35,62,63]. TSQ's derivative, Zinquin (*K*_d_: 620 nM) was developed to increase cellular retention [64]. However, both TSQ and Zinquin were found to coordinate non-labile Zn^2+^, that is pre-bound with proteins [60,65] and low molecular weight ligands (glutamic acid, glutathione, histidine and ATP) [66]. Recently, a pH insensitive sensor SpiroZin2 (*K*_d_: 3.6 nM) was used to detect vesicular Zn^2+^ in hippocampal mossy fibres [68] and lysosomal Zn^2+^ in lactating mouse mammary epithelial cells [51].

Synapse activity causes Zn^2+^ to release along with neurotransmitters, subsequently increasing extracellular Zn^2+^ concentration in the synaptic cleft. Direct quantitative measurement of such brief (within a few milliseconds) [122] and localized Zn^2+^ signals within the synaptic cleft remains challenging, but a large body of evidence has detected synaptically released Zn^2+^ and estimated that extracellular Zn^2+^ can increase from 1–20 nM baseline concentration to high micromolar concentration [34,78,98,123–126]. The cell membrane impermeable version of Zn^2+^ sensors such as FluoZin-3 (*K*_d_: 15 nM), ZP4 (*K*_d_: 0.65 nM) and ZnAF-2 (*K*_d_: 2.7 nM) have been used to examine Zn^2+^ release at hippocampal mossy fibre synapses [34,42,46,71–77,101,126,127]. By utilizing different affinities of sensors ZnAF-2 and ZnAF-3 (*K*_d_: 790 nM), it has been demonstrated that membrane depolarization induces differential amounts of Zn^2+^ release in the hippocampus, with the highest in the dentate gyrus compared to CA1 and CA3 [34]. Cell impermeable Newport Green DCF has low affinity for Zn^2+^ (K_d_: 1 µM), making it ideal for detecting high concentrations of Zn^2+^, and it has been used to visualize vesicular Zn^2+^ release in the hippocampal hilus [78,79]. The ratiometric Zn^2+^ sensor LZ9 (*K*_d_ = 0.57 nM) was developed for more precise quantification of extracellular Zn^2+^ to correct for the varieties in tissue thickness, sensor concentrations and imaging acquisitions [82]. LZ9 was designed by linking a green Zn^2+^ sensor with lissamine rhodamine B (LRB), which is a Zn^2+^-insensitive red fluorophore [82,83]. With LZ9, the extracellular Zn^2+^ concentration in the mice dorsal cochlear nucleus (DCN) was measured and detected under electrical stimulations [82].

## 3. Synaptic Zn^2+^ signalling in the brain

**Figure 1.**
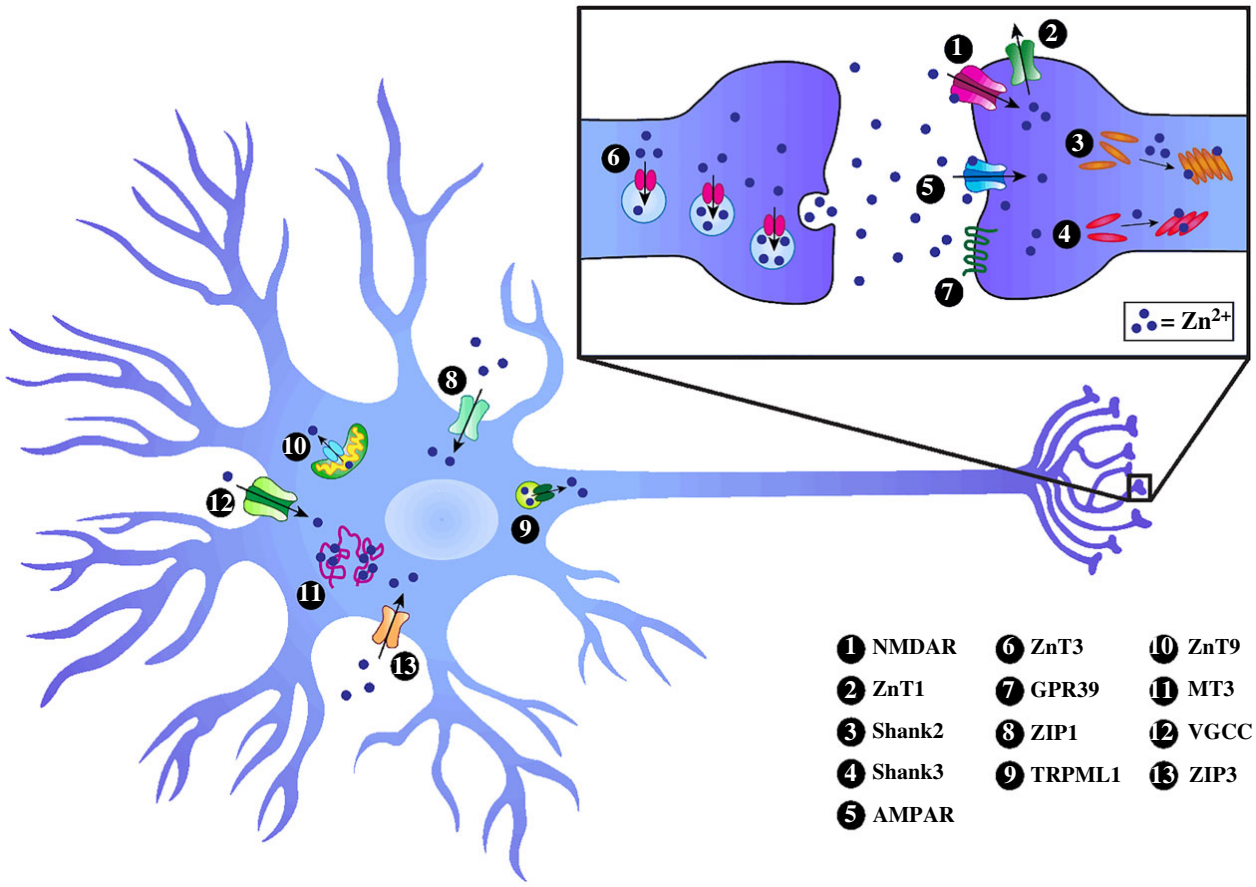
Modulation and targets of intracellular and synaptic Zn^2+^ signals in glutamatergic neurons. Intracellular Zn^2+^ signals can be generated via influx from extracellular environments mediated by ZIP1, ZIP3, and opening of ion channels (NMDAR, GluA2-lacking AMPAR, VGCC), liberation from metallothioneins (MT3), or release through the TRPML1 channel from lysosomes and late endosomes. Cytosolic Zn^2+^ is transported out of neurons by ZnT1 and sequestered into synaptic vesicles by ZnT3. Low concentrations of mitochondrial Zn^2+^ are maintained by ZnT9. Synaptically released Zn^2+^ can inhibit AMPAR containing GluA2 subunits and NMDAR to regulate synaptic activity. Zn^2+^ can also induce GPR39-mediated signalling. Postsynaptic Zn^2+^ signals translocate Shank2 and Shank3 to postsynaptic regions, thereby enhancing recruitments of AMPAR's GluA2 subunits and promoting removal of GluA1 subunits.

The presence of abundant Zn^2+^ in the synaptic vesicles of zincergic neurons has been confirmed by histochemical staining [29,31,97–100,128], electron microscopy [111], and microscopy imaging using the fluorescent sensors discussed in the previous section. This pool of Zn^2+^ is concentrated into synaptic vesicles through vesicular transporter ZnT3 [129,130] (figure 1), and co-released with neurotransmitters, such as glutamate, to the synaptic cleft during physiological neuronal excitation [31,101,131]. Synaptically released Zn^2+^ has been suggested to act via phasic mode, which refers to the situation that free Zn^2+^ immediately increases in the synaptic cleft, diffuses away from released sites and acts on target cells [118,132–135]. There is evidence that such synaptic Zn^2+^ can diffuse into extrasynaptic regions during repetitive synaptic stimulations [82]. Synaptically released Zn^2+^ acts as an intercellular signalling molecule to regulate activity of presynaptic or postsynaptic neurons, astrocytes and microglia cells [118,133–135] by targeting a variety of ionotropic and metabotropic receptors located on cells. The neurobiological roles of synaptic Zn^2+^ have been investigated extensively by three strategies: (a) eliminating Zn^2+^ inside synaptic vesicles using ZnT3 knockout mice created by Dr Richard Palmiter's team [130], (b) chelating extracellular Zn^2+^ with membrane-impermeable chelators such as CaEDTA, Tricine and ZX1[84,136–140], or (c) mutating the Zn^2+^ binding sites on the receptor proteins such as glycine receptor alpha1 subunit [140] and glutamate receptor GluN2A subunit [98]. In addition to the transient high concentrations of synaptic Zn^2+^, low concentrations of ambient extracellular Zn^2+^ (less than 10 nM) might also act as a signalling molecule via tonic mode. Such ambient Zn^2+^ was suggested to derive from accumulation of synaptically released Zn^2+^ that is still coordinated with membrane proteins [141], or from efflux of cytoplasmic Zn^2+^ in postsynaptic cells by Zn^2+^ transporter ZnT1 [142] (figure 1). Different results were reported regarding whether tonic Zn^2+^ affects cell excitability, which might be due to the differences in the synapses chosen to study, Mg^2+^ concentration in the experimental buffers, or chelators used [82,98].

A number of ionotropic receptors can be regulated by Zn^2+^ including N-methyl-D-aspartate receptors (NMDARs), α-amino-3-hydroxy-5-methyl-4-isoxazole propionic acid receptors (AMPARs) without GluA2 subunit [143], gamma-aminobutyric acid receptors (GABARs), glycine receptors, L-type and N-type voltage-gated calcium channels (VGCCs), voltage-gated sodium channels, voltage-gated potassium channels, P2X purinergic receptors and transient receptor potential ankyrin 1 (TRPA1) channels [144–149] (figure 1). Zn^2+^ displays differential modulation (inhibition, excitation or activation) of these targets with varying potency depending on target isoform types, Zn^2+^ concentration and synaptic activity [138]. For example, NMDAR subtypes containing GluN2A subunits possess a nanomolar-affinity Zn^2+^ binding site, while GluN2B subunits have a micromolar-affinity site for Zn^2+^ [150–152]. For AMPAR, hundreds of micromolar Zn^2+^ demonstrates inhibition towards the AMPAR containing GluA2 subunits [153]. By tuning the gating of these ion channels, synaptically released Zn^2+^ modulates excitatory and inhibitory postsynaptic currents (EPSCs and IPSCs respectively), and hence synaptic activity [120,121,126,140,154]. The effects of synaptic Zn^2+^ on EPSCs have been studied in brain regions that are rich in glutamatergic Zn^2+^, including hippocampal mossy fibre-CA3, hippocampal Schaffer collateral-CA1 synapses and DCN parallel fibre synapses, where it was discovered that presynaptic stimulation discharges Zn^2+^ that inhibits NMDAR EPSCs [11,82,98,109] and AMPAR ESPCs [84]. In addition, synaptically released Zn^2+^ inhibits GABA_A_R IPSCs in principal neurons in the lateral amygdala [120], but enhances GABA_A_R IPSCs in somatostatin interneurons in the auditory cortex [155] as well as glycinergic IPSCs in hypoglossal motoneurons of the brainstem [140,156,157].

Emerging studies also revealed the roles of synaptic Zn^2+^ in regulating metabotropic receptors. Hershfinkel's group demonstrated that synaptically released Zn^2+^ from mossy fibre stimulation interacts with a ‘Zn^2+^-sensing receptor’ (ZnR/GPR39) on postsynaptic cells in CA3 of hippocampal slices (figure 1), which triggers the G protein-coupled receptor pathway and subsequently induces the release of Ca^2+^ from thapsigargin-sensitive intracellular pools [118,158]. Such Zn^2+^-evoked Ca^2+^ signals induce a series of MAPK-dependent cascades, promoting interaction between SNAP23 and K^+^/Cl^−^ cotransporter 2 (KCC2), enhancing the surface expression and activity of KCC2 in hippocampal neurons [158,159]. The increased KCC2-mediated Cl^−^ outward current maintains the hyperpolarizing GABA_A_R reversal potentials, reducing glutamate excitotoxicity in hippocampal neurons. This has further been supported by the finding that GPR39 knockout mice are more susceptible to kainate-induced seizures compared to wild-type groups [160]. The ZnR was also reported to be present in the DCN, where activation of ZnR by synaptic Zn^2+^ promotes synthesis of endocannabinoids, resulting in the reduction of presynaptic glutamate release in a retrograde manner [113]. However, ZnR/GPR39 has very low binding affinity for Zn^2+^ (*K*_d_: 150 µM), raising the question of whether it acts as a physiological receptor of Zn^2+^. Early studies have suggested that Zn^2+^ exerts effects via tropomyosin receptor kinase B (TrkB), through either activating metalloprotease to increase mature brain-derived neurotropic factor (BDNF) [161], or transactivating TrkB directly [154]. However, this hypothesis was challenged by inconsistent results in ZnT3 knockout mice. Although several studies showed that there is an increased level of BDNF in ZnT3 knockout mice [162–165], the TrkB protein levels in ZnT3 knockout mice have been reported to be increased [165], downregulated [162,163], or unchanged [164,166].

Through modulating the activity of the vast array of receptors, the signals of synaptic Zn^2+^ are transduced to impact brain function. For example, synaptically released Zn^2+^ facilitates long-term potentiation (LTP) at the cortico-amygdala synapses via depressing feedforward GABAergic inhibition of principal neurons [120]. Partially due to the roles of synaptic Zn^2+^ in amygdala synaptic plasticity, ZnT3 knockout mice showed deficiency in subtle and complex learning of fear [167]. Synaptic Zn^2+^ in the hippocampus was suggested to enhance LTP in the CA1 region [168] and modulate the mossy fibre LTP in the CA3 regions [11,109]. As hippocampal LTP is the basis of normal cognitive function, synaptic Zn^2+^ might be involved in learning and memory [77,169]. ZnT3 knockout mice showed mild deficits in long-term memory and spatial memory [170,171]. In addition, synaptically released Zn^2+^ in the auditory cortex and somatosensory cortex aids in sensory processes as ZnT3 knockout mice have been shown to have deficits in distinguishing different sound frequencies [136,172] and detecting fine textural differences [173].

## 4. Modulation and function of intracellular Zn^2+^ signals in neurons

Dynamic changes in intracellular Zn^2+^ concentration are tightly regulated in neurons by a multitude of membrane transporters, ion channels and buffering proteins, which control Zn^2+^ signals both spatially and temporally. The Zn^2+^ transporters include 10 efflux transporters (ZnTs, SLC30A) and 14 influx transporters (ZIPs, SLC39A). ZnTs extrude Zn^2+^ into the extracellular space or intracellular compartments [31,129,130,174,175], while ZIPs allow Zn^2+^ inflow by or coupled with H^+^ or HCO_3_^−^ gradients [176–179]. The expression of Zn^2+^ transporters has distinct localization and roles in various brain areas and subcellular compartments. ZnT1 (SLC30A1), which is primarily localized on the plasma membrane [180–182], was shown to be closely tied with NMDA receptors at postsynaptic densities [183] (figure 1). ZnT3 (SLC30A3) is localized on the membrane of synaptic vesicles and highly co-localized with synaptic Zn^2+^ [31,129,130,174,175] (figure 1). ZnT9 (SLC30A9) is a mitochondrial Zn^2+^ transporter which maintains low Zn^2+^ inside mitochondria [184] (figure 1). TMEM163 is another Zn^2+^ transporter expressed in the synaptic vesicles and has been categorized as ZnT11 [185]. Both ZIP1 and ZIP3 are ubiquitous plasma membrane transporters, but they display distinct expressions in the hippocampus [178,186] (figure 1). ZIP1 is enriched in the stratum pyramidale of CA3, while ZIP3 is highly expressed in dentate gyrus granule cells [187]. ZIP4 has high expression in the soma and postsynaptic regions of Purkinje neurons in the cerebellum [188].

Metallothioneins (MTs) are a family of cysteine-rich (30%) proteins, including four human isoforms (MT1, MT2, MT3, MT4), which play critical roles in regulating gene expression, controlling cellular metal metabolism and adjusting cellular adaptation to stress [189]. The MT3 protein consists of two subdomains (*α* and *β* domain), and has the capacity of binding up to seven Zn^2+^ ions [190] (figure 1). MT3 is the brain-specific MT, responsible for buffering cellular Zn^2+^ in neurons and astrocytes [191–194]. The high amounts of cysteine residues in MTs are accessible to modification by reactive oxygen or nitrogen species, thus liberating Zn^2+^ to the cytosol [195–197]. Compared to MT1 and MT2, MT3 demonstrated higher reactivity to release more Zn^2+^ when treated with S-nitrosothiols due to the enhanced nitrosylation of multiple cysteines adjacent to basic and acid residues in MT3 [198]. Accordingly, exogenous nitric oxide increases intracellular Zn^2+^ in neurons [199]. In addition, cytosolic acidification following Ca^2+^ influx has been suggested to induce intracellular Zn^2+^ release in neurons [48,49].

Labile Zn^2+^ is maintained at nanomolar concentration (approx. 20 nM) in cerebrospinal fluid (CSF) in the normal brain [200], while the concentration of Zn^2+^ can reach up to 15 000 fold (approx. 300 µM) in the synaptic cleft under both spontaneous and electrically stimulated conditions [109,179,200,201]. It is still unclear whether the synaptically released Zn^2+^ can flux into neurons, but immediate increases in cellular Zn^2+^ can be mediated through opening of ion channels on the plasma membrane or lysosomal membrane. Both fluorescence-based imaging techniques and electrophysiology have provided strong evidence that activated voltage-gated Ca^2+^ channels are permeable to Zn^2+^ in mammalian neurons [147,149,202–204]. Recent electrophysiology and Zn^2+^ fluorescent imaging studies show that some TRP subfamilies are Zn^2+^ permeable [205]. TRPA1 channels, which are mainly located in dorsal root ganglia neurons, are permeable to Zn^2+^ [146]. Another TRP channel, transient receptor potential canonical type 6 (TRPC6), demonstrates Zn^2+^ permeability in mouse cortical neurons and contributes to nuclear Zn^2+^ accumulation [206]. In addition, the lysosomal channel transient receptor potential mucolipin 1 (TRPML1) mediates Zn^2+^ release from late endosomes in primary rat neuron culture [52] (figure 1).

Increases in cellular Zn^2+^ signals might act similarly to Ca^2+^ signals, mediating a cascade of phosphorylation signalling events that involve protein kinases and proteases. The enzymes that can be regulated by intraneuronal Zn^2+^ should have a half-maximal inhibitory concentration (IC_50_) within physiological range of Zn^2+^ concentrations (high picomolar to low nanomolar). Several enzymes demonstrate this high Zn^2+^ binding affinity *in vitro* including tyrosine phosphatase beta activity (IC_50_ = 21 pM) [207], caspase 3 (IC_50_ < 10 nM) [208], and Ca^2+^-ATPase (IC_50_ = 80 pM) [209]. Tyrosine phosphatases are expressed in neurons and play a role in modifying synaptic formation as well as neuronal development [210]. Intraneuronal Zn^2+^ release, mediated by nitric oxide, was found to activate p38 MAPK, resulting in apoptosis in cortical neurons [199]. Zn^2+^ reuptake into the hippocampal mossy fibre terminal was suggested to inhibit MAPK tyrosine phosphatase, thereby inducing Erk activation [211]. However, a recent study in HeLa cells and mouse hippocampal neuronal cell line (HT-22) suggests that nanomolar Zn^2+^ activates Erk and Akt signalling pathways via the upstream molecule Ras, while activation of Erk phosphatase requires a Zn^2+^ concentration higher than is reached by physiological fluctuations [212]. In addition, members of the MAPK pathway proteins (e.g. MAPK1, MAPK4, Fibroblast growth factor receptor 3, Fibroblast Growth Factor Receptor Substrate 2, Rap guanine nucleotide exchange factor 2, c-Jun) were also upregulated at the transcriptional level by increases in intraneuronal Zn^2+^ [213].

The Shank proteins are another target of intraneuronal Zn^2+^ signals and the interaction between Zn^2+^ and Shank proteins regulates synapse formation and function. The Shank proteins, a family of scaffolding proteins located in the excitatory synapses of neurons, consist of three members (Shank1, Shank2 and Shank3) [214,215] (figure 1). Shank protein is a ‘master’ scaffolding protein, interacting and coordinating with intermediate scaffolding proteins at the postsynaptic regions [214]. Shank proteins indirectly interact with NMDA receptors by linking PSD95 to guanylate kinase (GK)-associated proteins [216,217]. There is a direct interaction between AMPAR's GluA1 subunit and Shank3's PDZ domain [218]. In addition, Shank assists synaptic growth and maturation through affecting the internalization of the transmembrane Wnt receptor Frizzled-2 (Fz2) [219]. It has been well studied that internalization and cleavage of Fz2 play an important role in modulating synapse development [220,221]. Among the three members, Shank2 and Shank3 contain a sterile-alpha-motif domain at their C-terminal that can bind Zn^2+^, while Shank1 is insensitive to Zn^2+^ [222,223]. Binding of Zn^2+^ is required for oligomerization and assembly of both Shank2 and Shank3 [223,224], essential for their proper postsynaptic localization during synaptogenesis and synapse maturation [225]. The Zn^2+^ sensitive Shank proteins are expressed before Shank1 in neurons, resulting in a difference in Zn^2+^ sensitivity between young neurons and mature neurons [225]. Neuron depolarization-induced Zn^2+^ signals interact with Shank2 and Shank3, recruiting AMPAR GluA2 subunits and removing GluA1 subunits at the glutamatergic synapses, hence enhancing AMPAR synaptic efficacy in young neurons [224,226] (figure 1). In mouse models of Zn^2+^ deficiency, Shank protein levels were significantly reduced in the striatum, hippocampus, cortex and cerebellum and the mice displayed developmental and behavioural issues mimicking autism spectrum disorders [227].

## 5. Conclusion

The surge in development of a full suite of new fluorescent Zn^2+^ sensors, chelators and genetic mice models allows us to gain a greater understanding about the neuronal signalling of Zn^2+^ in live primary neuron culture, brain slices and live animals. The dynamic changes in neuronal Zn^2+^ signals have been evidenced by the detection of extracellular Zn^2+^ signals during synaptic activity and intracellular Zn^2+^ signals during influx, neuron excitation and oxidative stress. Simultaneous measurement of intracellular Zn^2+^ concentrations and signalling molecules in live cells has confirmed that physiologically relevant Zn^2+^ dynamics regulate Erk signalling events. Behavioural studies in live mice with the application of fast and selective Zn^2+^ chelators along with genetic knockdown of ZnT3 further elucidated the involvement of synaptic Zn^2+^ signals in learning, memory, emotion, sensory function and social interaction.

However, it is still not completely unveiled when, where and how physiological changes in intracellular and intercellular Zn^2+^ signals can regulate their targets. We still lack tools that are sensitive and bright enough to track localized Zn^2+^ signals within synapses and neurons in whole organisms, preventing us from tackling many of these unanswered questions. Another challenge is that occurrence of Zn^2+^ signals is accompanied with the changes in other cellular signals such as Ca^2+^, pH and redox potential, which adds to the complexity of investigating the Zn^2+^ signalling roles. New sensors are needed to resolve these challenges. In addition, given that there are multiple zincergic neurons and Zn^2+^ targets, the function of synaptic Zn^2+^ signals in a specific brain region is hard to clarify from whole-body ZnT3 knockout mice. Conditional knockout of ZnT3 in specific brain regions and neuron types will further elucidate the roles of synaptic Zn^2+^ in the brain.

## Data Availability

This article has no additional data.
